# The Vulnerability to Methamphetamine Dependence and Genetics: A Case-Control Study Focusing on Genetic Polymorphisms at Chromosomal Region 5q31.3

**DOI:** 10.3389/fpsyt.2022.870322

**Published:** 2022-05-20

**Authors:** Jing Xiao, Yitian Ma, Xiaochen Wang, Changqing Wang, Miao Li, Haobiao Liu, Wei Han, Huiying Wang, Wenpei Zhang, Hang Wei, Longrui Zhao, Tianxiao Zhang, Huali Lin, Fanglin Guan

**Affiliations:** ^1^Department of Forensic Medicine, School of Medicine & Forensics, Xi’an Jiaotong University, Xi’an, China; ^2^Department of Health Science, Chang’an Drug Rehabilitation Center, Xi’an, China; ^3^Department of Ultrasound, The Second Affiliated Hospital, Xi’an Jiaotong University, Xi’an, China; ^4^Department of Epidemiology and Biostatistics, School of Public Health, Xi’an Jiaotong University, Xi’an, China; ^5^Department of Psychiatry, Xi’an Mental Health Center, Xi’an, China

**Keywords:** methamphetamine dependence, genetic polymorphisms, case-control study, HDAC3, Han Chinese

## Abstract

**Objectives:**

Methamphetamine (METH) is a central nervous psychostimulant and one of the most frequently used illicit drugs. Numerous genetic loci that influence complex traits, including alcohol abuse, have been discovered; however, genetic analyses for METH dependence remain limited. An increased histone deacetylase 3 (*HDAC3*) expression has been detected in Fos-positive neurons in the dorsomedial striatum following withdrawal after METH self-administration. Herein, we aimed to systematically investigate the contribution of *HDAC3* to the vulnerability to METH dependence in a Han Chinese population.

**Methods:**

In total, we recruited 1,221 patients with METH dependence and 2,328 age- and gender-matched controls. For genotyping, we selected 14 single nucleotide polymorphisms (SNPs) located within ± 3 kb regions of *HDAC3*. The associations between genotyped genetic polymorphisms and the vulnerability to METH dependence were examined by single marker- and haplotype-based methods using PLINK. The effects of expression quantitative trait loci (eQTLs) on targeted gene expressions were investigated using the Genotype-Tissue Expression (GTEx) database.

**Results:**

The SNP rs14251 was identified as a significant association signal (χ^2^ = 9.84, *P* = 0.0017). An increased risk of METH dependence was associated with the A allele (minor allele) of rs14251 [odds ratio (95% CI) = 1.25 (1.09–1.43)]. The results of *in silico* analyses suggested that SNP rs14251 could be a potential eQTL signal for *FCHSD1*, *PCDHGB6*, and *RELL2*, but not for *HDAC3*, in various human tissues.

**Conclusion:**

We demonstrated that genetic polymorphism rs14251 located at 5q31.3 was significantly associated with the vulnerability to METH dependence in Han Chinese population.

## Introduction

Methamphetamine (METH), a well-known powerful central nervous psychostimulant, is currently the most commonly used illicit drug in China (1). METH can cause damage to multiple organs, such as the heart, gut, and brain (2). Meanwhile, individuals with a history of chronic METH use are likely to develop METH-related psychosis, including auditory hallucinations and paranoid thinking (3). METH dependence is a huge financial burden on people with addiction and their families. In addition, it can provoke a series of violent events, leading to several social problems. Although the precise mechanism underlying METH dependence remains unknown, METH-induced changes in gene expression were shown to be closely related to severe dysregulation of normal neurophysiological brain activity. More recently, genome-wide association studies have been extensively employed to detect correlations between genetic variants and complex diseases, such as schizophrenia, coronary heart disease, and height in samples from various populations (4, 5). Numerous loci that influence complex traits, such as alcohol and other substance abuse, have been identified, facilitating our understanding of underlying molecular mechanisms (6–8). However, data on METH dependence remains scarce despite multiple reports of candidate genes (9).

Epigenetic alterations can lead to persistent structural chromatin adaptations, indicating that epigenetic modifications may play a critical role in the METH-induced gene expression changes (10, 11). Furthermore, abnormal microRNA expression is known to mediate METH dependence (12). Histone deacetylases (HDACs) are proteins involved in histone acetylation and can be classified into four classes according to the homologous similarity of their sequences. Class I, including HDAC1, HDAC2, HDAC3, and HDAC8, is considered critical for transcriptional repression and epigenetic modulation (13). As HDAC3 is highly expressed in the adult brain, its effects on transcriptional regulation related to learning and memory have attracted considerable attention (14). In the METH-induced conditioned place preference (CPP) model, an increased histone 3 acetylation was identified in the limbic forebrain of mice and found in specific gene-promoter regions related to synaptic plasticity, such as Nrxn, Gria1, Grin2a, and Grin2b, thus indicating its essential role in METH dependence (15). An increased expression of HDAC3 mRNA has been noted in Fos-positive neurons in the dorsomedial striatum after withdrawal following METH self-administration (16). Studies have shown that sodium butyrate, a non-selective inhibitor of class I/II HDACs, can help overcome a previously established CPP and suppress the reinstatement of METH-induced CPP (17). These findings suggest that HDAC3 may be a key molecule for regulating METH-associated gene expression and clarifying the underlying biological mechanism. However, the relationship between the *HDAC3* gene and METH dependence remains elusive. In this study, we aimed to systematically explore the risk susceptibility of HDAC3 to METH dependence among Han Chinese population.

## Materials and Methods

### Study Subjects

Herein, we enrolled 1,221 patients with METH dependence and 2,328 age-matched healthy controls from the Chang’an Drug Rehabilitation Center of Xi’an City and the Second Affiliated Hospital of Xi’an Jiaotong University, respectively. All participants were genetically unrelated individuals of Han Chinese origin (at least three generations were of Han descent and had no history of migration). The inclusion criteria for the METH dependence group were as follows: (1) 11 criteria for substance use disorders (Supplementary Table 1) according to Diagnostic and Statistical Manual of Mental Disorders-Fifth Edition (DSM-V); (2) METH use >2 days per week for >1 year; (3) no use disorders (DSM-V criteria) considering other addictive substances, including alcohol and marijuana. Participants with tumors, neurodegenerative disorders, and other severe organic disorders were excluded from the study. In addition, participants were excluded if they met the criteria for past or current manic episodes, schizophrenia, schizoaffective disorder, or other psychotic disorders based on the DSM-V. Peripheral blood samples were collected from participants and preserved for further genotyping experiments. Demographic characteristics of all participants were collected using questionnaires and are presented in Table 1. All participants provided written informed consent. The study procedures were approved by the Medical Ethics Committee of Xi’an Jiaotong University Health Science Center and performed in accordance with the ethical guidelines of the Declaration of Helsinki (version 2013).

**TABLE 1:** Sociodemographic characteristics of the study subjects.

Variables	Patients with METH dependence (*N* = 1,221)	Controls (*N* = 2,328)	Statistics	*P*-Value
Age, years	32.2 ± 6.3	32.2 ± 6.5	*t* = −0.047	0.96
**Gender (%)**				
*Males*	748 (61)	1,425 (61)		
*Females*	473 (39)	903 (39)	χ^2^ < 0.001	1.00
**Employment status (%)**				
*Yes*	673 (55)	2144 (92)		
*No*	548 (45)	184 (8)	χ^2^ = 666.67	<0.01
**Marital status (%)**				
*Married*	454 (37)	1755 (75)		
*Single or devorced*	767 (63)	573 (25)	χ^2^ = 495.79	<0.01

*METH, methamphetamine. Age (continuous variable) is presented as mean ± standard deviation (SD).*

### Candidate Single Nucleotide Polymorphisms Selection and Genotyping

Single nucleotide polymorphisms with a minor allele frequency (MAF) >0.05 in mixed population data and located within ± 3 kb regions of *HDAC3* were selected. This strategy formed a set of 22 SNPs. We excluded 5 SNPs that were non-polymorphic in the 1000 Genomes database for the Han Chinese population. In addition, we excluded 3 indels; accordingly, 14 candidate SNPs were selected for further genotyping experiments (Supplementary Table 2). Peripheral blood was drawn from each participant, and genomic DNA was extracted using a commercial DNA kit according to the manufacturer’s protocol (Axygen Scientific Inc., Union City, CA, United States). SNP genotyping experiments were conducted using the Sequenom MassARRAY platform. The raw data were processed, and the genotypic data were released using the Typer Analyzer. Technicians involved in experimental processes were blinded to case or control labels. A small portion of study samples (5%) were randomly selected for replication experiments to assess the accuracy of SNP genotyping.

### Statistical Analyses

To estimate the statistical power of the study, a power analysis was implemented using the Genetic Association Study (GAS) Power Calculator.^1^ The power analysis results are summarized in Supplementary Figure 1. The power analysis results indicated that the sample size level was sufficient to detect a SNP with moderate effect. The Hardy-Weinberg equilibrium (HWE) tests were performed for genotyping quality control based on obtained data for control individuals. The associations between genotyped genetic polymorphisms and vulnerability to METH dependence were examined using single marker- and haplotype-based methods. Both allelic and genotypic distributions of genetic polymorphisms in participants with METH dependence and healthy controls were determined using PLINK (18) for single marker-based association analyses. Linkage disequilibrium (LD) patterns of the 14 candidate genetic polymorphisms were defined based on a standard algorithm (19). Visualization of the LD structure was achieved using the Haploview version 4.2 (20). The statistical significance of the association analyses was examined using χ^2^ tests. Multiple testing was performed using the Bonferroni corrections. The *P*-value threshold was set as 0.05 divided by the number of independent tests.

### Bioinformatics Analyses

We examined the effects of expression quantitative trait loci (eQTLs) on targeted gene expressions using the Genotype-Tissue Expression (GTEx) database (21), which integrates genetic polymorphism information and gene expression data from various types of human tissues to depict the patterns of human genome eQTLs. Sorting Intolerant From Tolerant (SIFT) (22) and Polymorphism Phenotyping v2 (Polyphen-2) (23) were utilized to explore the potential functional consequences of non-synonymous DNA variants. Both tools are designed to predict the impact of amino acid changes on the structure and function of the protein. The protein-protein interaction (PPI) networks of *HDAC3* were explored using the Search Tool for the Retrieval of Interacting Genes/Proteins (STRING) version 11.5 database (24). STRING is a publicly available database of PPIs stemming from computational prediction or knowledge obtained from functional studies.

## Results

### Demographic and Characteristic Features of Participants

A total of 1,221 patients with METH dependence and 2,328 controls were enrolled. No significant differences were observed in terms of age and gender between patients and controls, as both variables were matched at enrollment (Table 1). We noted significant differences in employment and marital status between the patients with METH dependence and controls. In addition, certain socioeconomic status (SES) variables were distinctly distributed among the two groups.

### Association Between Genetic Polymorphisms of HDAC3 and Methamphetamine Dependence

**FIGURE 1:**
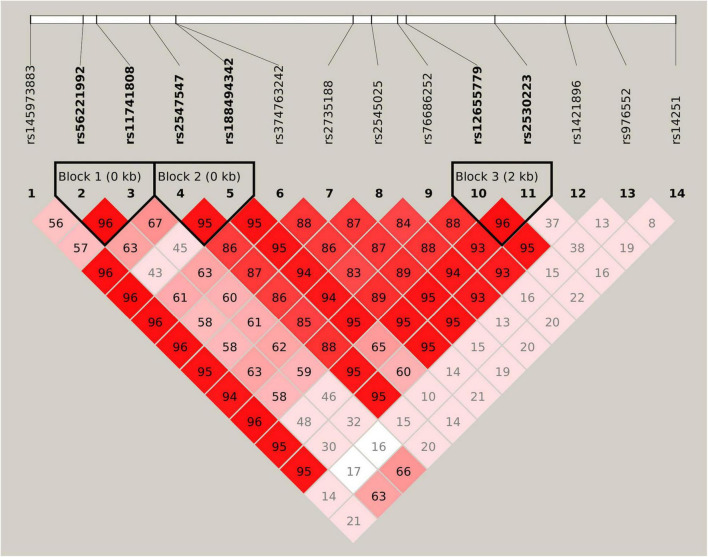
Linkage disequilibrium (LD) structure of 14 selected single nucleotide polymorphisms (SNPs). Values of D’ are indicated in each cell. The LD blocks are shown in thick-lined boxes, and SNPs of the haplotype blocks are highlighted in bold font.

All 14 SNPs showed HWE in the control group, and the results are presented in Supplementary Table 2. The SNP rs14251 was identified as a significant signal in the association analyses (Table 2), and its *P*-value was significant in allelic analyses following Bonferroni correction (χ^2^ = 9.84, *P* = 0.0017, Supplementary Table 3). Nominal significance was observed at the genotype level of this SNP (χ^2^ = 10.37, *P* = 0.0056). An increased risk of METH dependence was associated with the A allele (minor allele) of rs14251. The odds of exhibiting a copy of A allele vs. C allele was 25% higher in patients with METH dependence than in controls [odds ratio (OR): 1.25; 95% CI: 1.09–1.43]. Three LD blocks were constructed and visualized, as shown in Figure 1. The haplotype-based analyses revealed significant differences in haplotypic frequencies between patients and controls in the rs56221992-rs11741808 LD block (Table 3). Notably, a significant difference was observed between the two groups for the TA haplotype (χ^2^ = 47.89, *P* = 4.50 × 10^–11^).

**TABLE 2:** Results of association analyses of SNP rs14251 and METH dependence.

SNP	Position	Type of analyses	Group	Patients with METH dependence (*N* = 1,221)	Controls (*N* = 2,328)	OR (95% CI)	χ^2^	*P*-Value
rs14251	5:141639543	Genotypic analyses	AA (%)	28 (2)	43 (2)	1.33 (0.82–2.15)		
			AC (%)	327 (27)	518 (22)	1.29 (1.10–1.51)		
			CC (%)	866 (71)	1767 (76)	ref	10.37	0.0056
		Allelic Analyses	A (%)	383 (16)	604 (13)	1.25 (1.09–1.43)		
			C (%)	2059 (84)	4052 (87)	ref	9.84	0.0017

*SNP, single nucleotide polymorphism; METH, methamphetamine; OR, odds ratio; CI, confidence interval. The “ref” indicates the reference group.*

**TABLE 3:** Results of the haplotype-based association analyses.

Locus	SNPs	Length (kb)	Haplotype	F_A	F_U	χ^2^	DF	*P*-Value
**H1**	**rs56221992| rs11741808**	**0.345**	**OMNIBUS**	**–**	**–**	**48.630**	**2**	**2.75 × 10^–11^**
			TG	0.09	0.10	1.277	1	0.26
			**TA**	**0.03**	**0.01**	**47.890**	**1**	**4.50 × 10^–11^**
			CA	0.88	0.89	2.496	1	0.11
H2	rs2547547| rs188494342	0.667	OMNIBUS	–	–	0.698	2	0.71
			GG	0.10	0.09	0.409	1	0.52
			AG	0.08	0.08	0.354	1	0.55
			AA	0.83	0.83	0.005	1	0.94
H3	rs12655779| rs2530223	2.265	OMNIBUS	–	–	6.224	2	0.04
			GC	0.10	0.09	1.599	1	0.21
			AC	0.24	0.26	5.533	1	0.02
			AT	0.66	0.64	1.839	1	0.18

*SNP, single nucleotide polymorphism; F_A, haplotypic frequency in patients; F_U, haplotypic frequency in controls; DF, degree(s) of freedom. Significant results were highlighted in bold font.*

### Functional Consequences of Single Nucleotide Polymorphism rs14251

The data acquired from the GTEx database suggested that rs14251 is not involved in the expression level of *HDAC3* in any human tissue (Supplementary Table 4), including 13 brain tissues. Significant eQTL effects of this SNP were observed for other genes physically located around *HDAC3* (Table 4). These genes included *FCHSD1*, *PCDHGB6*, and *RELL2*. It should be noted that SNP rs14251 exerted varying effects on the expression levels of these genes. The A allele of SNP rs14251 increased the expression of *FCHSD1* and decreased the expression of both *PCDHGB6* and *RELL2*. Given that SNP rs14251 is located in the exonic region of *RELL2* and is a non-synonymous change, its functional consequence on this gene was predicted using SIFT and PolyPhen-2. SIFT indicated this SNP as “tolerated” and PolyPhen-2 as “benign,” respectively. The PPI network of *HDAC3* is depicted in Supplementary Figure 2.

**TABLE 4:** The effects of SNP rs14251 on target gene expressions that achieved *P*-Value < 1.00 × 10^–4^.

Gene	SNP	Ref_allele	Alt_allele	*P*-Value	NES	Tissue
*FCHSD1*	rs14251	C	A	9.90 × 10^–8^	0.12	Artery–Tibial
*FCHSD1*	rs14251	C	A	4.10 × 10^–6^	0.10	Adipose–Subcutaneous
*FCHSD1*	rs14251	C	A	1.00 × 10^–5^	0.10	Cells–Cultured fibroblasts
*FCHSD1*	rs14251	C	A	1.30 × 10^–5^	0.09	Whole blood
*FCHSD1*	rs14251	C	A	3.80 × 10^–5^	0.09	Nerve–Tibial
*FCHSD1*	rs14251	C	A	5.40 × 10^–5^	0.08	Lung
*PCDHGB6*	rs14251	C	A	7.90 × 10^–5^	−0.18	Esophagus–Muscularis
*RELL2*	rs14251	C	A	5.30 × 10^–25^	−0.36	Thyroid
*RELL2*	rs14251	C	A	1.90 × 10^–7^	−0.29	Spleen
*RELL2*	rs14251	C	A	1.10 × 10^–6^	−0.14	Esophagus–Mucosa
*RELL2*	rs14251	C	A	1.20 × 10^–6^	−0.16	Breast–Mammary Tissue
*RELL2*	rs14251	C	A	3.20 × 10^–6^	−0.14	Skin–Not sun exposed (Suprapubic)
*RELL2*	rs14251	C	A	1.00 × 10^–5^	−0.12	Whole blood
*RELL2*	rs14251	C	A	4.30 × 10^–5^	−0.14	Brain –Caudate (basal ganglia)
*RELL2*	rs14251	C	A	6.20 × 10^–5^	−0.09	Brain–Cortex
*RELL2*	rs14251	C	A	8.70 × 10^–5^	−0.16	Artery–Aorta
*RELL2*	rs14251	C	A	9.90 × 10^–5^	−0.11	Skin–Sun exposed (Lower leg)

*SNP, single nucleotide polymorphism; Ref_allele, reference allele; Alt_allele, alternative allele; NES, normalized effect size.*

## Discussion

A variety of neurotransmitter and receptor system-related genes, including *BDNF*, *DRD2*, and *GABRB2*, were found to contain genetic variants that contribute to the vulnerability of METH use disorder (9). Although multiple lines of evidence have associated epigenetic alterations with METH dependence (25, 26), no relevant population-based studies focusing on epigenetic-related genes have been conducted. To the best of our knowledge, this study is the first to link DNA variants of HDAC3 and vulnerability to METH dependence in a population-based study. Recently, Rudzinskas et al. have indicated that METH alters HDAC and DNA methyltransferase (DNMT) activity in the posterior dorsal medial amygdala of rats (26); therefore, our findings align with these results. Genetic markers of specific genes may modify the effects of METH on human epigenetic patterns.

Significant associations between haplotypes and vulnerability to METH dependence have been previously reported. Both SNPs rs56221992 and rs11741808 showed moderate levels of LD with rs14251 (*r*^2^ = 0.3 for rs56221992 and *r*^2^ = 0.3 for rs11741808, respectively). Although we cannot exclude the possibility that this haplotypic association signal occurred by chance or originated from other underlying independent association signals, it could probably arise from SNP rs14251.

Single nucleotide polymorphism rs14251 is located in the 3′-untranslated region of *HDAC3*. It is also located in the exonic region of *RELL2* as a non-synonymous change. This double identity increases the complexity of predicting its functional consequences. Both SIFT and PolyPhen-2 predicted that this SNP exerts mild functional consequences on the protein structure of *RELL2*. Nevertheless, our *in silico* analyses revealed that SNP rs14251 could affect the expression levels of *RELL2*, *FCHSD1*, and *PCDHGB6* (but not that of *HDAC3*) in various kinds of human tissues. A recent study has linked *HDAC3* expression to schizophrenia (27). However, neither *RELL2* nor *FCHSD1* appears to be associated with psychiatric or brain-related traits. *RELL2* reportedly encodes receptors expressed in lymphoid tissues such as 2 protein, which participates in the positive regulation of the p38MAPK cascade and is speculated to be a human tumor necrosis factor (28). *FCHSD1* encodes F-BAR protein and double SH3 domains protein 1. A recent study by Kawasaki et al. has indicated that the loss of *FCHSD1* could ameliorate chronic obstructive pulmonary disease (29). In addition, the third gene, *PCDHGB6*, encodes a calcium-dependent cell-adhesion protein, potentially related to the activity of specific neuronal connections in the human brain (30). However, this study results are insufficient to functionally map SNP rs14251 to any of the four genes. Therefore, it might be more appropriate to map this SNP to the 5q31.3 genomic region instead of a specific gene.

Although eQTL data in the GTEx database indicated that SNP rs14251 could be mapped to the three surrounding genes, the results of *in silico* analyses should be cautiously interpreted. First, SNP rs14251 is related to gene expressions in various kinds of human tissues; however, 13 types of brain tissues (e.g., cortex, cerebellar, and others) are not listed. In other words, METH-dependence targeted tissues are not included. In addition, the METH addiction status for patients whose data were obtained from the GTEx database remains largely unknown. A gene expression pattern has specific spatial and temporal features, and it may considerably differ between patients and healthy participants. Accordingly, animal-based functional studies remain crucial to properly map SNP rs14251 to its functionally relevant gene and unravel potential consequences.

The PPI network constructed in this study indicates that *HDAC3* interacts with several other genes for implementing its biochemical and biological functions. Among the genes included in this network, *PPARG* is of particular interest. *PPARG* is known to encode peroxisome proliferator-activated receptor (PPAR) gamma, which is a member of the PPARs, a subfamily of nuclear receptors (31). A recent genome-association study has linked *PPARG* genetic polymorphisms to human cognitive measurements (32). As METH-use disorders are widely associated with cognitive functions, this study highlights the complexity of pathogenic mechanisms underlying METH dependence.

In addition, the limitations of this study need to be addressed. Although we have selected SNPs located at the gene region of *HDAC3* with MAF >0.05, the genetic information coverage may be insufficient. Recent studies on several psychiatric disorders, including bipolar disorder and nicotine dependence, have indicated that genetic variants with low frequency might substantially contribute to the susceptibility of relevant disorders (33, 34). In the future, low-frequency and rare DNA variants should be explored to exhaustively examine the genetic contribution of *HDAC3* to the vulnerability to METH dependence. Replication studies are also needed to rule out the “winner’s curse.” In addition, the controls recruited in this study might not be comparable with METH-dependent individuals. To examine the vulnerability to METH dependence, ideal controls would be those individuals who use METH but are not addicted; this, in turn, might affect the association measures.

## Conclusion

In summary, we demonstrated that genetic polymorphism rs14251 located at 5q31.3 was significantly associated with the vulnerability to METH dependence in a Han Chinese population.

## Data Availability Statement

According to national legislation/guidelines, specifically the Administrative Regulations of the People’s Republic of China on Human Genetic Resources (http://www.gov.cn/zhengce/content/2019-06/10/content_5398829.htm, http://english.www.gov.cn/policies/latest_releases/2019/06/10/content_281476708945462. htm), no additional raw data is available at this time. Data of this project can be accessed after an approval application to the China National Genbank (CNGB, https://db.cngb.org/cnsa/). Please refer to https://db.cngb.org/, or email: CNGBdb@cngb.org for detailed application guidance. The accession code CNP0002872 should be included in the application.

## Ethics Statement

The studies involving human participants were reviewed and approved by the Medical Ethics Committee of Xi’an Jiaotong University Health Science Center. The patients/participants provided their written informed consent to participate in this study.

## Author Contributions

FG, TZ, and HLin conceived and designed the study. JX performed the computational analyses and a candidate SNP selection and wrote the first draft of the manuscript. YM and XW performed the statistical analyses of genotype and haplotype data. CW, ML, WH, and HLiu conducted the study subjects’ phenotypic screening. HLiu, HWe, WZ, HWa, and LZ contributed to the collections and preparations of control subjects’ DNA samples. All authors revised the current manuscript.

## Conflict of Interest

The authors declare that the research was conducted in the absence of any commercial or financial relationships that could be construed as a potential conflict of interest.

## Publisher’s Note

All claims expressed in this article are solely those of the authors and do not necessarily represent those of their affiliated organizations, or those of the publisher, the editors and the reviewers. Any product that may be evaluated in this article, or claim that may be made by its manufacturer, is not guaranteed or endorsed by the publisher.

## Footnotes

1
https://csg.sph.umich.edu/abecasis/gas_power_calculator
