# Supplementary material for: The Vulnerability to Methamphetamine Dependence and Genetics: A Case-Control Study Focusing on Genetic Polymorphisms at Chromosomal Region 5q31.3
Source: Front Psychiatry. 2022 May 20;13:870322. doi: 10.3389/fpsyt.2022.870322 (PMC9163382; doi:10.3389/fpsyt.2022.870322)
Supplement: Supplementary file 1 [file Data_Sheet_1.docx]

**Supplementary Tables**

**Supplementary Table S1.** The 11 criteria for substance use disorders.

| **No.** | **Categories of Behavior** | **Criteria for Substance Use Disorders (SUD)*** |
| --- | --- | --- |
| 1 | Impaired control | Used larger amounts or longer: Taking the drug in greater quantities or over prolonged periods of time |
| 2 |  | Repeated attempts to control use and/or quit: Wanting to cut or avoid using the substance, but they haven't been successful |
| 3 |  | Much time spent using: Spending a lot of time to get, using, or recover from substance using |
| 4 |  | Craving: Cravings and encourages the substance to be used |
| 5 | Social impairment | Activities given up to use: Not able to do what you can do at home, at work, or at school that you once liked because of substance use |
| 6 |  | Social or interpersonal problems related to use: Continuing to use, even though it creates issues in your relationships or conflicts with others |
| 7 |  | Neglected major roles to use: Giving up and refusing to perform significant social, occupational or recreational functions as a result of substance use |
| 8 | Risky use | Hazardous use: Using substances again and again, including though you or others are in danger |
| 9 |  | Social or interpersonal problems related to use: Continuing to use, even though you know that you have a physical or psychological condition which may have been triggered or exacerbated by the substance |
| 10 | Pharmacological indicators | Tolerance: Need more substance to have the effect you like |
| 11 |  | Withdrawal: Development of withdrawal symptoms and signs of withdrawal, which can be eased by taking more of the substance |

**Supplementary Table S2.** Genetic information and results for Hardy-Weinberg equilibrium tests of the 14 selected SNPs.

| CHR | Position | Locus | SNP | Function | Minor Allele | Major Allele | MAF | P_HWE1_ | P_HWE2_ |
| --- | --- | --- | --- | --- | --- | --- | --- | --- | --- |
| 5 | 141623077 | *HDAC3* | rs145973883 | intron | A | C | 0.11 | 0.66 | 0.32 |
| 5 | 141624420 | *HDAC3* | rs56221992 | intron | T | C | 0.11 | 0.78 | 0.91 |
| 5 | 141624764 | *HDAC3* | rs11741808 | intron | G | A | 0.10 | 0.17 | 0.49 |
| 5 | 141626120 | *HDAC3* | rs2547547 | intron | G | A | 0.10 | 0.43 | 1.00 |
| 5 | 141626786 | *HDAC3* | rs188494342 | intron | G | A | 0.17 | 0.84 | 0.77 |
| 5 | 141626788 | *HDAC3* | rs374763242 | intron | G | A | 0.09 | 0.52 | 0.90 |
| 5 | 141631309 | *HDAC3* | rs2735188 | intron | C | T | 0.10 | 0.21 | 0.34 |
| 5 | 141631779 | *HDAC3* | rs2545025 | intron | C | A | 0.10 | 0.09 | 0.72 |
| 5 | 141632446 | *HDAC3* | rs76686252 | intron | A | G | 0.08 | 0.09 | 0.65 |
| 5 | 141632663 | *HDAC3* | rs12655779 | intron | G | A | 0.10 | 0.13 | 1.00 |
| 5 | 141634927 | *HDAC3* | rs2530223 | coding-synonymous | C | T | 0.35 | 0.52 | 0.96 |
| 5 | 141636721 | *HDAC3* | rs1421896 | intron | G | T | 0.32 | 0.90 | 0.74 |
| 5 | 141637772 | *HDAC3* | rs976552 | intron | C | A | 0.16 | 0.75 | 0.65 |
| 5 | 141639543 | *HDAC3* | rs14251 | untranslated-3 | A | C | 0.14 | 0.75 | 0.46 |

SNP, single nucleotide polymorphism; CHR, chromosome; MAF, minor allele frequency; HWE, Hardy-Weinberg equilibrium. P_HWE1_: *P* value for HWE tests conducted in cases; P_HWE2_: *P* value for HWE tests conducted in controls.

**Supplementary Table S3.** Detailed results of the single marker-based association analyses.

| CHR | SNP | A1 | A2 | TEST | AFF | UNAFF | χ^2^ | DF | *P* |
| --- | --- | --- | --- | --- | --- | --- | --- | --- | --- |
| 5 | rs145973883 | A | C | GENO | 16/236/969 | 30/428/1870 | 0.477 | 2 | 0.788 |
| 5 | rs145973883 | A | C | ALLELIC | 268/2174 | 488/4168 | 0.410 | 1 | 0.522 |
| 5 | rs56221992 | T | C | GENO | 15/256/950 | 26/437/1865 | 2.600 | 2 | 0.273 |
| 5 | rs56221992 | T | C | ALLELIC | 286/2156 | 489/4167 | 2.408 | 1 | 0.121 |
| 5 | rs11741808 | G | A | GENO | 15/198/1008 | 26/413/1889 | 1.363 | 2 | 0.506 |
| 5 | rs11741808 | G | A | ALLELIC | 228/2214 | 465/4191 | 0.769 | 1 | 0.380 |
| 5 | rs2547547 | G | A | GENO | 15/218/988 | 21/401/1906 | 1.113 | 2 | 0.573 |
| 5 | rs2547547 | G | A | ALLELIC | 248/2194 | 443/4213 | 0.749 | 1 | 0.387 |
| 5 | rs188494342 | G | A | GENO | 35/353/833 | 67/671/1590 | 0.003 | 2 | 0.998 |
| 5 | rs188494342 | G | A | ALLELIC | 423/2019 | 805/3851 | 0.001 | 1 | 0.973 |
| 5 | rs374763242 | G | A | GENO | 14/213/994 | 18/384/1926 | 1.839 | 2 | 0.399 |
| 5 | rs374763242 | G | A | ALLELIC | 241/2201 | 420/4236 | 1.365 | 1 | 0.243 |
| 5 | rs2735188 | C | T | GENO | 17/216/988 | 26/399/1903 | 0.709 | 2 | 0.701 |
| 5 | rs2735188 | C | T | ALLELIC | 250/2192 | 451/4205 | 0.547 | 1 | 0.460 |
| 5 | rs2545025 | C | A | GENO | 19/217/985 | 22/394/1912 | 3.132 | 2 | 0.209 |
| 5 | rs2545025 | C | A | ALLELIC | 255/2187 | 438/4218 | 1.948 | 1 | 0.163 |
| 5 | rs76686252 | A | G | GENO | 13/177/1031 | 14/316/1998 | 2.931 | 2 | 0.231 |
| 5 | rs76686252 | A | G | ALLELIC | 203/2239 | 344/4312 | 1.925 | 1 | 0.165 |
| 5 | rs12655779 | G | A | GENO | 19/223/979 | 21/402/1905 | 3.759 | 2 | 0.153 |
| 5 | rs12655779 | G | A | ALLELIC | 261/2181 | 444/4212 | 2.376 | 1 | 0.123 |
| 5 | rs2530223 | C | T | GENO | 134/557/530 | 292/1068/968 | 2.286 | 2 | 0.319 |
| 5 | rs2530223 | C | T | ALLELIC | 825/1617 | 1652/3004 | 2.031 | 1 | 0.154 |
| 5 | rs1421896 | G | T | GENO | 129/540/552 | 237/1001/1090 | 0.843 | 2 | 0.656 |
| 5 | rs1421896 | G | T | ALLELIC | 798/1644 | 1475/3181 | 0.734 | 1 | 0.392 |
| 5 | rs976552 | C | A | GENO | 28/327/866 | 65/631/1632 | 0.869 | 2 | 0.648 |
| 5 | rs976552 | C | A | ALLELIC | 383/2059 | 761/3895 | 0.517 | 1 | 0.472 |
| 5 | rs14251 | A | C | GENO | 28/327/866 | 43/518/1767 | 10.370 | 2 | 0.006 |
| 5 | rs14251 | A | C | ALLELIC | 383/2059 | 604/4052 | 9.836 | 1 | 0.002 |

SNP, single nucleotide polymorphism; CHR, chromosome; A1, tested allele (minor allele); A2, major allele; GENO, genotypic; AFF, counts for genotypes or alleles for patients; UNAFF, counts for genotypes or alleles for controls; DF, degree(s) of freedom.

**Supplemental Table S4.** The effects of SNP rs14251 on target gene *HDAC3*’s expression

| Gene | SNP | Ref_allele | Alt_  allele | *P*-Value | NES | T-statistic | Tissue |
| --- | --- | --- | --- | --- | --- | --- | --- |
| *HDAC3* | rs14251 | C | A | 0.004 | 0.0800 | 2.90 | Esophagus - Mucosa |
| *HDAC3* | rs14251 | C | A | 0.015 | 0.0960 | 2.50 | Testis |
| *HDAC3* | rs14251 | C | A | 0.035 | -0.1100 | -2.10 | Brain - Frontal Cortex (BA9) |
| *HDAC3* | rs14251 | C | A | 0.037 | 0.1100 | 2.10 | Pituitary |
| *HDAC3* | rs14251 | C | A | 0.066 | 0.0400 | 1.80 | Skin - Not Sun Exposed (Suprapubic) |
| *HDAC3* | rs14251 | C | A | 0.110 | 0.0360 | 1.60 | Cells - Cultured fibroblasts |
| *HDAC3* | rs14251 | C | A | 0.110 | -0.0400 | -1.60 | Muscle - Skeletal |
| *HDAC3* | rs14251 | C | A | 0.150 | 0.0820 | 1.50 | Cells - EBV-transformed lymphocytes |
| *HDAC3* | rs14251 | C | A | 0.230 | -0.1000 | -1.20 | Brain - Amygdala |
| *HDAC3* | rs14251 | C | A | 0.230 | 0.0390 | 1.20 | Breast - Mammary Tissue |
| *HDAC3* | rs14251 | C | A | 0.250 | -0.0530 | -1.20 | Heart - Atrial Appendage |
| *HDAC3* | rs14251 | C | A | 0.290 | 0.0230 | 1.10 | Whole Blood |
| *HDAC3* | rs14251 | C | A | 0.300 | 0.0220 | 1.00 | Skin - Sun Exposed (Lower leg) |
| *HDAC3* | rs14251 | C | A | 0.310 | -0.0780 | -1.00 | Ovary |
| *HDAC3* | rs14251 | C | A | 0.310 | 0.0460 | 1.00 | Spleen |
| *HDAC3* | rs14251 | C | A | 0.330 | -0.0490 | -0.98 | Adrenal Gland |
| *HDAC3* | rs14251 | C | A | 0.350 | -0.0370 | -0.93 | Heart - Left Ventricle |
| *HDAC3* | rs14251 | C | A | 0.390 | 0.0490 | 0.87 | Artery - Coronary |
| *HDAC3* | rs14251 | C | A | 0.410 | -0.0450 | -0.83 | Brain - Hypothalamus |
| *HDAC3* | rs14251 | C | A | 0.440 | -0.0420 | -0.78 | Small Intestine - Terminal Ileum |
| *HDAC3* | rs14251 | C | A | 0.450 | 0.0320 | 0.76 | Pancreas |
| *HDAC3* | rs14251 | C | A | 0.490 | 0.0180 | 0.68 | Adipose - Subcutaneous |
| *HDAC3* | rs14251 | C | A | 0.520 | -0.0420 | -0.64 | Minor Salivary Gland |
| *HDAC3* | rs14251 | C | A | 0.520 | 0.0400 | 0.64 | Prostate |
| *HDAC3* | rs14251 | C | A | 0.530 | 0.0290 | 0.63 | Brain - Cortex |
| *HDAC3* | rs14251 | C | A | 0.540 | -0.0270 | -0.61 | Colon - Sigmoid |
| *HDAC3* | rs14251 | C | A | 0.570 | -0.0220 | -0.57 | Brain - Nucleus accumbens (basal ganglia) |
| *HDAC3* | rs14251 | C | A | 0.570 | 0.0160 | 0.57 | Lung |
| *HDAC3* | rs14251 | C | A | 0.590 | 0.0260 | 0.54 | Brain - Putamen (basal ganglia) |
| *HDAC3* | rs14251 | C | A | 0.670 | -0.0140 | -0.43 | Nerve - Tibial |
| *HDAC3* | rs14251 | C | A | 0.680 | -0.0310 | -0.42 | Brain - Substantia nigra |
| *HDAC3* | rs14251 | C | A | 0.680 | 0.0160 | 0.41 | Colon - Transverse |
| *HDAC3* | rs14251 | C | A | 0.720 | 0.0130 | 0.36 | Artery - Aorta |
| *HDAC3* | rs14251 | C | A | 0.720 | -0.0140 | -0.35 | Brain - Cerebellar Hemisphere |
| *HDAC3* | rs14251 | C | A | 0.740 | -0.0110 | -0.33 | Thyroid |
| *HDAC3* | rs14251 | C | A | 0.760 | -0.0170 | -0.31 | Brain - Hippocampus |
| *HDAC3* | rs14251 | C | A | 0.770 | -0.0210 | -0.30 | Brain - Anterior cingulate cortex (BA24) |
| *HDAC3* | rs14251 | C | A | 0.800 | 0.0110 | 0.25 | Stomach |
| *HDAC3* | rs14251 | C | A | 0.820 | -0.0073 | -0.23 | Adipose - Visceral (Omentum) |
| *HDAC3* | rs14251 | C | A | 0.840 | -0.0064 | -0.20 | Artery - Tibial |
| *HDAC3* | rs14251 | C | A | 0.880 | -0.0074 | -0.15 | Brain - Cerebellum |
| *HDAC3* | rs14251 | C | A | 0.880 | 0.0110 | 0.15 | Brain - Spinal cord (cervical c-1) |
| *HDAC3* | rs14251 | C | A | 0.880 | 0.0100 | 0.16 | Vagina |
| *HDAC3* | rs14251 | C | A | 0.890 | -0.0100 | -0.13 | Uterus |
| *HDAC3* | rs14251 | C | A | 0.960 | -0.0015 | -0.05 | Esophagus - Muscularis |
| *HDAC3* | rs14251 | C | A | 0.990 | 0.0003 | 0.01 | Brain - Caudate (basal ganglia) |
| *HDAC3* | rs14251 | C | A | 0.990 | 0.0007 | 0.02 | Liver |

SNP, single nucleotide polymorphism; Ref_allele, reference allele; Alt_allele, alternative allele; NES, normalized effect size; EBV, Epstein-Barr virus.

**Supplementary Figures**

**
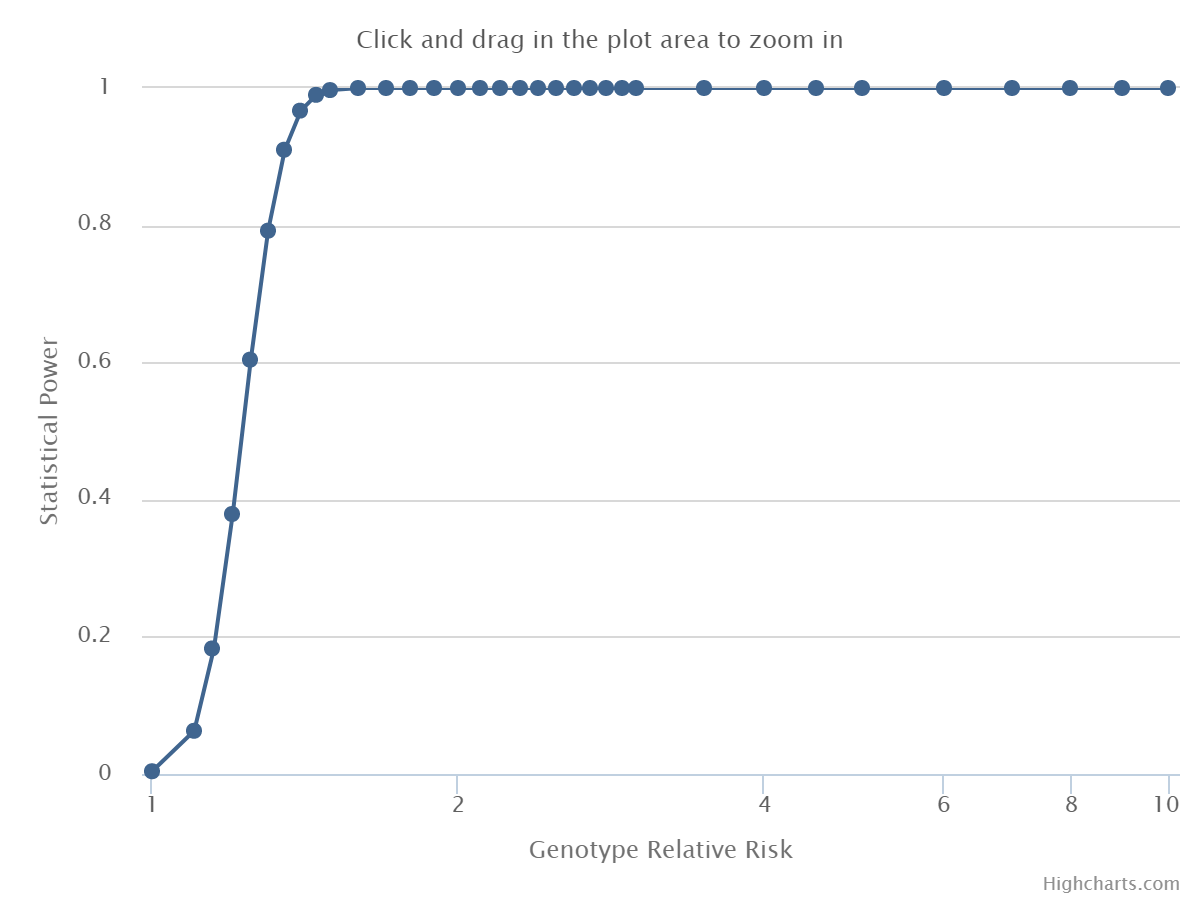
**

**Power=79.2%, RR=1.30**

**Supplementary Figure S1.** Statistical power as a function of genotype relative risk (RR).


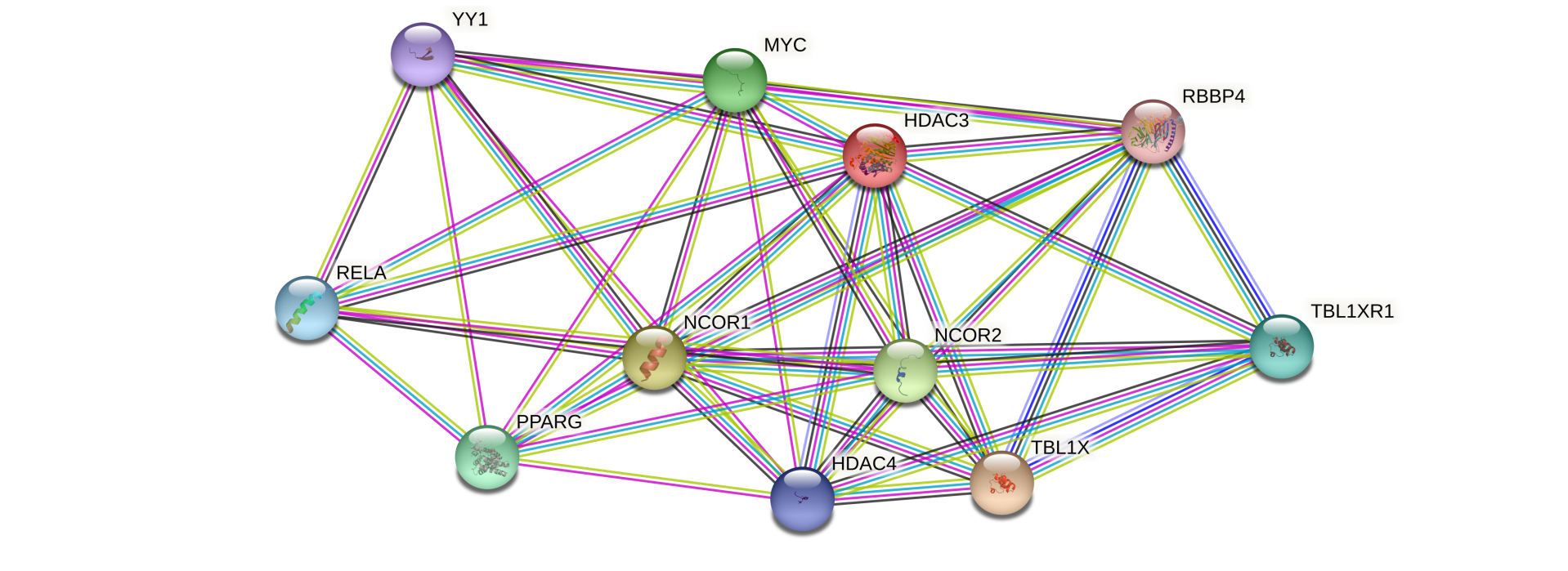


**Supplementary Figure S2.** Protein-protein interaction (PPI) network based on *HDAC3* constructed by using STRING.
